# Supplementary material for: Feasibility and acceptability for LION, a fully remote, randomized clinical trial within the VA for light therapy to improve sleep in Veterans with and without TBI: An MTBI2 sponsored protocol
Source: PLoS One. 2025 Jan 7;20(1):e0305305. doi: 10.1371/journal.pone.0305305 (PMC11706480; doi:10.1371/journal.pone.0305305)
Supplement: S1 File — (PDF) [file pone.0305305.s002.pdf]

Research Protocol

Title:
Improving sleep, sleep-related outcomes, and biomarkers in Veterans.
#4268

Investigators:
Principal Investigator: Miranda Lim, MD, PhD

Specific Aims/Purpose:
The primary purpose of this project is to determine the effect of morning bright light therapy (MBLT) on sleep, cognition, pain, and other measures of quality of life in Veterans with and without traumatic brain injury (TBI). Secondarily, the project aims to identify blood-based brain biomarkers (BBBM) associated with sleep, cognition, and pain in Veterans. Subjects will be recruited across two sites, Oregon Health & Science University (OHSU) and the VA Portland Health Care System (VAPORHCS), with OHSU as the coordinating center.

Specific Aim 1. Determine the effect of MBLT on objective and subjective sleep quality in Veterans.

Specific Aim 2. Determine the effect of MBLT on downstream effectors of improved sleep, including cognition, pain, and quality of life measures in Veterans.

Specific Aim 3. Determine the effect of MBLT on levels of specific BBBM related to sleep, cognition, and pain, and whether changes in specific BBBM predict response to MBLT.

Hypothesis: We hypothesize that MBLT will improve sleep, cognition, pain, and quality of life in Veterans with TBI, thereby resulting in an improvement in related BBBM.

Scientific Rationale and Significance:
Statement of Problem. Estimated rates of mild traumatic brain injuries (TBI) among returning military personnel from recent combat engagements have ranged from 12%-15% in Veterans surveyed following their return home [1, 2] and up to 59% in an at-risk group of injured military personnel receiving trauma care at Walter Reed Army Hospital [3]. Persistent sleep disruption following a TBI is one of the most common complaints, which is present in 50-70% of individuals who sustain a TBI [4]. Sleep disruption has been associated with impaired learning, memory, and may contribute to prolonged post-concussive symptoms after TBI, suggesting that sleep disruption may contribute to the high rates of psychiatric and neurological symptoms and deficits following a TBI. Sleep disruption has also been implicated in the risk for neurodegeneration following TBI. Specifically, disturbed sleep has been linked to a greater risk of developing Alzheimer's disease (AD) [5]. Pre-clinical studies have linked sleep disturbances to an increase in AD-related biomarkers, including amyloid beta (Aβ) and tau, suggesting that sleep is essential for clearance of these proteins, which is essential to reduce the risk for neuronal aggregations that contribute to plaques and tangles, the hallmark pathology of AD [6]. This is important as this may increase the risk for neurodegeneration, especially as our Veteran population with TBIs age and these risks may become more prominent. Therefore, treating sleep disturbances related to TBI may mitigate the neurodegenerative risks related to both sleep disruption and TBI [7-13]. Unfortunately, few effective treatment options exist for sleep disturbances following TBI. Cognitive-behavioral therapy for insomnia (CBTi) has the most evidence base for treating insomnia in TBI [14-16]; however, it is resource intensive and has high attrition rates [17, 18]. Medications such as sedative-hypnotics for sleep are often ineffective and discontinued due to side effects and/or tolerance. Thus, there is an urgent need to identify simple, yet effective treatment options. Furthermore, objective biomarkers of TBI-associated sleep disruption are needed to reveal the underlying pathophysiology and track response to therapy.

Blood-based biomarkers represent a practical and non-invasive approach for biospecimen analyses, especially among individuals with closed head TBIs and those who do not have cerebrospinal fluid (CSF) available for analyses. While peripheral blood samples are nearly ubiquitous in clinical TBI cohorts, one limitation of using circulating proteins from peripheral biomarkers is the inability to determine neuronal pathology, as proteins present in circulation may originate from peripheral sources. To address this limitation, our collaborators at the Military Traumatic Brain Injury Initiative (MTBI^2), formerly known as the Center for Neuroscience and Regenerative Medicine (CNRM), at the NIH have developed the ability to isolate brain derived exosomes, allowing examination of central processes that relate to sleep from peripheral blood. However, more research is needed to identify new TBI-associated sleep biomarkers. As we have an increasing number of survivors of TBI in our Veteran population, we are faced with the growing problem of providing effective rehabilitation for individuals who have experienced TBI. By improving sleep alongside measurement of biomarkers, we aim to reduce the burden of TBI in our military Veterans.

Proposed Solution. This project will utilize a novel method to affect sleep and a novel approach to measuring biomarkers in Veterans. We will use morning bright light therapy (MBLT) to improve sleep. Light is the primary timekeeper of the circadian system. Light facilitates entrainment of rhythms in affected circadian rhythm disorders through activation of intrinsic photoreceptors in retinal ganglion cells that project to the brain's master clock, the suprachiasmatic nucleus, via the retinohypothalamic tract [19-23]. This light activation required for regular sleep-wake cycles, and incorrect use of light increases risk for poor quality sleep, including development of insomnia [23-26]. Pre-clinical models have shown that light exposure alone can affect sleep, weight, and physical activity [27-29]. MBLT has been successfully used to improve sleep in individuals with depression and dementia, effects that have been shown to be independent of circadian effects [24, 30-33]. Studies have shown direct effects of MBLT on mood symptoms, presumably through projections to limbic brain areas that control mood [34-37]. Thus, functional improvement after MBLT is likely to have pleiotropic effects, involving direct changes to sleep, circadian entrainment, and mood.

MBLT is a simple, cost-effective, home-based sleep intervention. It has been studied in a variety of neuropsychiatric populations with similar features to TBI, such as seasonal affective disorder, non-seasonal depression, Alzheimer's, Huntington's, Parkinson's disease, and schizophrenia [32, 38-45]. MBLT is an attractive intervention for TBI as patients typically report multiple concomitant symptoms including alterations in mood and alertness (daytime fatigue). One recent placebo-controlled study using MBLT reported improved fatigue in individuals with TBI [46]. Moreover, MBLT has high patient acceptability and is scalable, owing to its ability to be rapidly implemented in any setting including in the home or during travel.

To address the substantial limitation of using peripheral biomarkers to understand central pathophysiology in TBI, we have developed a method to use blood-based brain biomarkers (BBBM) to track central pathophysiology and symptomatology of TBI and response to treatment. This method entails isolation of neuronally derived exosomes in peripheral blood. Exosomes are membrane boundmu structures that, instead of being delivered to lysosomes for destruction, reside in multivesicular bodies, fuse with the plasma membrane, and are exocytosed into extracellular spaces [47]. Within the membrane of exosomes are various proteins present in the cell of origin; thus, the cargo of exosomes reflects the microenvironment from the site of exosome production. Exosomes hold promise in TBI since they can readily cross the blood brain barrier (BBB) and be isolated from peripheral circulation. Studying exosomes allows us to isolate those proteins that are routinely disrupted in patients with TBI and sleep disturbances, and to track changes in accordance with treatment condition over time.

In this project, we will conduct a randomized controlled, single blinded trial in which Veterans and Non-Veterans with and without TBI will receive MBLT or sham (modified negative ion generator; *see below for modification specifications*) for 4-weeks. BBBM will be assessed in conjunction with MBLT/sham and neurobehavioral symptomatology. We hypothesize that: **1) MBLT will be associated with improved sleep, and downstream effectors of improved sleep (i.e., cognition, pain, and**

quality of life), compared to the sham condition; 2) MBLT will show a correlation between BBBM and improved sleep, and levels of BBBM will predict those that respond to MBLT. This will provide novel insights into how these biomarkers relate to neuronal and behavioral changes following TBI and may inform trajectory of recovery.

Preliminary Studies:

We have conducted several studies that highlight the need for this project and inform experimental design. Our preliminary data has examined; 1) the prevalence of sleep disturbances in Veterans – a cohort which identifies a large, immediately available pool of subjects for recruitment for this study, 2) the feasibility of the MBLT intervention in Veterans with sleep disturbances, and 3) the feasibility and promise of examining BBBM in individuals with TBI and sleep disorders.

MIRB #3641 and 3636

Title: *Factors that affect response to treatment for obstructive sleep apnea and related sleep disorders*

Publications:

Balba NM*, **Elliott JE***, Weymann KB, Opel RA, Duke JW, Oken BS, **Morasco BJ**, Heinricher MM, **Lim MM**. Increased sleep disturbances and pain in Veterans with comorbid TBI and PTSD. *Journal of Clinical Sleep Medicine*, 2018. *denotes equally contributing authors

Elliott JE, Opel RA, Papesh MA, Weymann KB, Chau AQ, Callahan ML, Storzbach D, **Lim MM**. Sleep disturbances in TBI: Associations with sensory sensitivity. *Journal of Clinical Sleep Medicine*, 2018.

Relevance:

This protocol recruited 670 Veterans across an ~3-year time frame from the VA Sleep Disorders Clinic, demonstrating our ability to access and recruit large numbers of Veterans with sleep problems.

MIRB #4085 and 4086

Title: *Morning bright light to improve sleep quality in Veterans*

Publications:

Data collection ongoing.

Relevance:

This protocol has recruited 91 participants over 18 months, 60 of which underwent MBLT for 4 weeks. The primary purpose of this protocol was to assess feasibility outcomes, including recruitment rate, retention rate, and adherence to the light therapy. Of the participants with TBI, 88% of subjects remained adherent to light therapy for the full 4 weeks. Overall, 91% of subjects said they liked using the lightbox. We additionally found that MBLT improves self-reported sleep, mood, and quality of life in our pilot cohort, however, this study lacks a placebo device for comparison.

Biomarker Studies at MBTI^2/NIH; Principal Investigator, Jessica Gill, PhD, RN

Publications:

Motamedi V, Kapogiannis D, **Gill J**. Exosomal Elevations of Tau and Amyloid-beta Relate to Chronic Symptoms Following a Traumatic Brain Injury, in NINR Advisory Council Meeting. 2016: Bethesda MD

Relevance:

Jessica Gill has developed advanced laboratory methods to isolate plasma exosomes enriched for neuronal origin, thereby providing insight into the central pathophysiology of TBI [65, 66]. Using these methods, we have completed the first clinical mild TBI study exploring exosomes and their cargo [67].

Research Design and Methods:
This is a multisite study in which subjects will be recruited from OHSU, VA Portland Healthcare System (VAPORHCS), and nationally in the community. For details regarding the informed consent process, inclusion/exclusion criteria, and specific recruitment strategies, see *Study Population* and *Subject Identification/Recruitment* sections below.

Participants who agree to participate in this study will be asked for their permission to include their study-related data and/or their contact information in a long-term data repository from which other research questions about sleep disorders, traumatic brain injuries, and functional outcomes might be studied. The data repository will be maintained by the Oregon Clinical and Translational Research Institute at Oregon Health & Science University (OHSU) in an online REDCap database (see below under Protected Health Information), with OHSU as the coordinating site.

This study will consist of at least 4 separate visits at VAPORHCS or completed remotely over the span of 10 weeks.

All visit activities may be completed remotely via phone or video conferencing and mailing of equipment with the exception of the blood draw. The blood draw is collected in person at the VA or at the participant's home, and may be skipped if saliva and sweat patch collection is performed.

Visit 1

- *Informed consent and HIPAA authorization*. Written and verbal informed consent will be obtained before all other Visit 1 activities and may take place on an earlier date if DocuSign is able to be used to facilitate the consent conversation. In cases where DocuSign is not feasible, consent conversations will take place in office space available at the VAPORHCS Sleep Disorders Clinic (building 100-6C), in related outpatient clinical space, in Research Service space approved for human subjects use (building 101, rooms 432A, 430, 404), at subject's homes, or remotely via mailed documents.
- *Demographics.* Participants will be asked to fill out demographics information which will include their name (including name at birth), date of birth, city/town of birth, email and physical address, phone number, health history, and military history.
- *Code of Conduct Handout*: The VA Portland Research Code of Conduct handout will be given to participants at the time of consent.
- Individuals without a VA medical record will be asked for permission for study staff to request their medical record to gather information about sleep, cognition, traumatic brain injury, and pain related issues.
- *Actigraphy*. Subjects will be asked to wear an individually configured actigraphy watch (Phillips Respironics Actiwatch 2) for the entirety of the study. This actiwatch collects data related to subject's sleep-wake cycle, including activity (via a built-in accelerometer) and light exposure (via a built-in luxometer). Watches are user friendly, requiring no user input to operate. Subjects will be reminded to keep clothing from covering up the luxometer on the actiwatch as much as possible.
- *Study diary*. Subjects will be asked to fill out a study diary, with daily entries for the entirety of the study. They will record when they went to bed, when they woke up, when they used the lightbox, and days they went to work. At this point subjects will be educated on principles of good sleep hygiene, such that all subjects start at the same baseline with respect to their understanding of good sleep hygiene principles. Study diaries will be coded via subject's ID with

no other identifiable information listed. The study diary may also be completed through RedCap survey links that are emailed or texted daily using the Twilio automated texting service.

- *Questionnaires*. Subjects will complete baseline questionnaires. They take ~30 min to complete and include past medical history and self-reported measures on sleep, social habits, quality of life, mental health, pain, and other metrics related to subject's prior trauma exposure history. All questionnaires are validated and will be completed via pen and paper or through electronic redcap surveys completed on a tablet, desktop, or laptop. Medical history and testing related to sleep will be accessed from the participants' VA medical records by study staff with VA credentials. Subjects may elect to complete these questionnaires at either this or subsequent visits as needed.
- *Light testing*. Stimulus-response functions for light will be determined for each subject using a photosensitivity tester (Bascom Palmer Eye Institute Photosensitivity Tester or remote option equivalent), which delivers a controlled stimulus and allows quantification of pupillary and blink responses. Participants report any discomfort with a button press and are always free to close their eyes if the light is perceived to be too bright. The light stimulation protocol will generally be with a preset pattern of flashes and interstimulus intervals. This testing takes ~7 minutes to complete. The extent of photosensitivity may depend on eye color. For example, in those with less pigmented eyes, the pupil size is not a good predictor of brightness because of the amount of light that passes through the iris. A photograph of the eyes alone (not full face) will be taken to control for this.
- *Department of Defense Head Trauma Events Checklist (DoD HTEC)*. Determination of TBI status occur through a semi-structured diagnostic interview following the DoD HTEC guidelines. This interview will take ~15 min to complete. Subjects may elect to complete this interview at either this or subsequent visits as needed.
- *Cognition testing*. Subjects will undergo a cognition assessment that includes domains related to memory, attention, executive function, language, and processing speed. The cognition assessment will take ~30 min. Subjects may elect to complete this testing at either this or subsequent visits as needed. In order to verify data, cognition testing may be audibly recorded on a digital voice recorder.
- *Blood draw*. Subjects will be directed to provide a blood sample. Phlebotomists will draw 2 Lavender top (appropriate for plasma separation) 10 ml tubes (or three 7 ml tubes), 2 Tiger top (appropriate for serum separation) 10 ml tubes (or three 7 ml tubes), and 2 PAX gene (appropriate for RNA/DNA analyses) 2.5 ml tubes. All samples will be immediately inverted ~10x. VA phlebotomy lab will notify study personnel when the samples are ready for pickup (1-2 min walk from our laboratory). On occasion, trained study personnel may draw the blood themselves in research service space (building 101 rooms 432A, 430, 404, or 533). Study personnel will spin down whole blood for the separation of plasma/serum. Aliquots will be stored at -80°C in a freezer behind a VA PIV badge secured research area (Building 101, 5th floor). Data obtained from these and other biosamples in this protocol will be used exclusively for research purposes with some samples being sent to MTBI^2 or other affiliate institutions or investigators like Dr. Jessica Gill at the NIH for analysis. Subjects may elect to complete this blood draw at either this or subsequent visits as needed.
- *Dim Light Melatonin Onset (DLMO) at home saliva collection*: Subjects may be asked to complete an at home saliva collection protocol in order to determine their DLMO. They will be sent home with 6 cotton plug "salivettes," and asked to chew on the salivettes until saturated with saliva once an hour for 6 hours starting 3 hours before their habitual bed time. Participants will be asked to store the used salivettes in their freezer until they can be mailed or brought into the VA..
- *Morning Cortisol at home saliva collection*: Subjects may be asked to complete at home saliva collection protocol in order to determine their morning cortisol levels. They will be sent home with 3-6 cotton plug salivettes and asked to saturate the salivettes with saliva every 15-30

minutes for 1.5 hours starting as soon as possible after waking. Participants will be asked to store the salivettes in the freezer until they can be mailed or brought into the VA.

- PharmChek Sweatpatch. Biomarkers including peripheral cytokines will be collected using a sweat patch that participants will be instructed to wear for 12-72 hours. They will be instructed to place it on their core and avoid being fully submerged in water (NB: showering is permissible). They will be mailed or brought to the VA where they will be stored in a -20 or -80 freezer until analysis. They will be coded and labeled with the subject's study ID and the timepoint. This may be mailed to the VA using standard DOT guidelines for biohazard shipment (e.g. triple containment) if the visits are completed remotely, or this can be completed at the VA.
- Oragene saliva collection. Saliva will be collected using the Oragene saliva collection kit for DNA analysis. Participants will provide about 2 mL of saliva into the Oragene tube that will then be labeled with subject ID and timepoint and stored at -80C in a locked freezer. This may be mailed to the VA using standard DOT guidelines for biohazard shipment (e.g. triple containment) if the visits are completed remotely, or this can be completed at the VA.
- Reimbursement. Subjects will be compensated $40 in gift cards or via ClinCards.
- These procedures may also take place at Visit 2, with only informed consent taking place at Visit 1. In that case, subjects will be compensated $40 at Visit 2 for completing the procedures/tests described above, and not compensated at Visit 1.
- Remote visits
    - In an effort to reduce subject burden of traveling to the Portland VA, we may try to do as many study requirements as possible remotely. Questionnaires and equipment may be mailed, and cognition testing and general instruction may take place over VA-approved video or phone conferencing (Skype, facetime, google hangouts, or VA Video connect).
        - In this scenario, consent would also take place over phone or video as follows:
            - Where DocuSign is feasible, study staff will initiate email of a DocuSign envelope providing subjects access to the consent and authorization forms ahead of the consent discussion. Subjects will then be guided through downloading the fully executed consent form.
            - Alternately, study personnel would mail the consent and authorization forms to them before hand and consent would be discussed and questions addressed over phone or video. Participants would then sign and mail back, and study personnel would sign when received. Study personnel would then mail them copies of the consent and HIPAA authorization forms.
    - Alternatively, if the participant prefers, study personnel may travel to subject's homes and conduct the visit in person there. Blood would be immediately transported back to the VA using tertiary containment. Questionnaires and consent forms would also be immediately brought back to the VA and placed in a locked cabinet.

Visit 2

- Visit 2 will occur ~2 weeks after Visit 1 and will take place remotely or within the VAPORHCS Sleep Disorders Clinic (building 100-6C), or in Research Service (building 101, rooms 432A, 430, 404, and 534).
- *Actigraphy*. Subjects will be given a new, fully charged/configured actiwatch and return their previous actiwatch.
- *Equipment*. Subjects will be randomized to receive a lightbox (LightPad mini, Aurora Light Solutions) or sham-control modified negative ion generator. Devices will be modified by completely disconnecting the electrical wiring required for negative ion generation. This will be verified using a voltmeter. A small fan may be installed to emit a low humming noise as an indicator of activity. They will be instructed to use either device for ~60 min every morning after

waking up for the next 4 weeks. Light sensitive devices, called HOBOs, will be attached to the lightboxes which will track when the lightbox is turned on or off, as a secondary measure of light delivery in addition to the luxometer on the actiwatch. Light testing equipment may also be mailed for remote visits.

- *Reimbursement*. The procedures described in Visit 1 (DLMO saliva collection, blood draw, cognition testing, HTEC, pain testing, and questionnaires) may be completed at Visit 2. If they are completed at Visit 2 instead of Visit 1, subjects will be compensated $40 in gift cards,via ClinCards or another cash equivalent. Depending on when the assays are completed, either Visit 1 or Visit 2 will be uncompensated.
- Remote option:
    - This visit may take place remotely, with equipment mailed and instructions discussed over the phone or video conferencing.
    - This visit may take place at the subject's home, with study personnel traveling to that location with the equipment. Saliva will immediately transported back to the VA using tertiary containment.

Visit 3

- Visit 3 will occur 4 weeks after Visit 2 and will take place remotely or within the VAPORHCS Sleep Disorders Clinic (building 100-6C), or in Research Service (building 101, rooms 432A, 430, 404, and 534).
- *Actigraphy*. Subjects will return the actiwatch, device, and sleep diary.
- *Measures*. The same series of questionnaires, light testing, cognition testing, and blood draw conducted at the baseline visit will be repeated at this, or future, visits.
- *Dim Light Melatonin Onset (DLMO) at home saliva collection*: Subjects may be asked to complete an at home saliva collection protocol in order to determine their DLMO.
- *Morning Cortisol at home saliva collection*: Subjects may be asked to complete an at home saliva collection protocol in order to determine their morning cortisol levels.
- *Sweat patch.* Subjects will provide a sweat sample as described in the baseline period.
- *Saliva sample.* Subjects will provide a saliva sample as described in the baseline period.
- *Reimbursement*. Subjects will be compensated $100 in gift cards,via ClinCard or another cash equivalent for completing the visit.
- Remote option:
    - In an effort to reduce subject burden of traveling to the Portland VA, we may try to do as many study requirements as possible remotely. Questionnaires may be mailed along with a return mailer for participant's to return equipment. Cognition testing may take place over video or phone conferencing.
    - This visit may take place at the subject's home. Blood, saliva, questionnaires, and equipment will be immediately transported back to the VA following the visit.

Visit 4 Follow-Up

- Subjects may be contacted for a follow-up visit occurring about 1 month after the intervention. This follow-up visit may include questionnaires, a quality assurance questionnaire, cognitive assessment, light testing and/or a blood-draw, morning cortisol saliva collection, sweat patch, and DLMO saliva collection. The reimbursement associated with this follow-up visit will be $40 in gift cards, via ClinCard or another cash equivalent.
- Questionnaires may be mailed along with a return mailer, and cognition testing may take place over phone or video conferencing.
    - This visit may take place at the subject's home. Blood, saliva, questionnaires, and equipment will be immediately transported back to the VA following the visit.

Phone calls. In between visits subjects will be contacted up to 2 times per week as needed. They will be assessed for understanding and compliance and given the opportunity to asked questions. Questions will be asked about their sleep, pain, cognition, and mood. The answers to the questions will be documented on paper and then entered into the MTBI^2 electronic data capture system Collection Access Sharing and Analytics (CASA) and OHSU Redcap database.

Mailed or emailed questionnaires. In between visits subjects may be mailed questionnaires to fill out and mail or bring back. They may also be emailed directly through RedCap or texted using a text messaging service.

Box contents/info sheets for remote visits: When equipment is shipped directly to the participant's home the box will include info sheets to describe and explain all the equipment in the box and what to do with them. They will also include dates of when to start/stop some activities.

Mailed equipment. In an effort to reduce subject's time at the Portland VA we may mail equipment and return envelopes directly to their homes. Instructions will be provided via phone or video conferencing.

Twilio automated texts. The sleep diary may be completed by daily RedCap surveys that are texted using the Twilio service. Twilio is a cloud based service that automates text messages. Twilio will access RedCap for a participant's phone number, but no other data will be stored. If participants indicate that they are open to receiving them, Twilio or other equivalent service may also be used to generate automated text message reminders by which subjects may confirm their upcoming study visits.

Video Conferencing: If the subject is comfortable and equipped, we may provide instructions on equipment use as well as cognition testing using a videoconferencing service, such as Skype for Business. A link to the video chat will be emailed participants. For VA Video Connect (VVC), this email is entered into the VCC platform and an email is automatically generated.

At home visits. In special circumstances study personnel may travel to the subject's homes in order to conduct the visit more comfortably. Equipment, data, and specimens will travel in between the home and the VA only.

Equipment management/tracking. Loaned equipment is tracked on a spreadsheet located on OHSU OneDrive or OHSU Dropbox, or future approved OHSU cloud storage services. This is true for mailed equipment and equipment physically handed to participants.

Overview:

<table>
<tr><th></th><th>Visit 1*</th><th>Visit 2**</th><th>At Home</th><th>Visit 3</th><th>Visit 4 Follow up</th></tr>
<tr><td>Time point:</td><td>Day 0</td><td>Week 2</td><td>Week 2-6</td><td>Week 6</td><td>Week 10</td></tr>
<tr><td>Informed consent</td><td>X</td><td></td><td></td><td></td><td></td></tr>
<tr><td>Subject given actiwatch</td><td>X</td><td>X</td><td></td><td>X</td><td></td></tr>
<tr><td>Subject given lightbox or sham</td><td></td><td>X</td><td></td><td>X</td><td></td></tr>
<tr><td>Questionnaires</td><td colspan="2">X</td><td></td><td>X</td><td>X</td></tr>
<tr><td>Cognition</td><td colspan="2">X</td><td></td><td>X</td><td>X</td></tr>
<tr><td>Sweat Patch</td><td colspan="2">X</td><td></td><td>X</td><td>X</td></tr>
<tr><td>Blood draw</td><td colspan="2">X</td><td></td><td>X</td><td>X</td></tr>
</table>

Salivary collection	X			X	X
Daily use of intervention for 1 hour			X		
**Total time**	**~120 min**	**~120 min**	**28 hours**	**~120 min**	**~120 min**

*The baseline questionnaires, cognition testing, blood draw and pressure pain testing will occur at either Visit 1 or Visit 2.
**Study visits should completed within 14 days from previous visit to remain within the visit window.

Study Population:

Number of subjects. The target population will consist of n=300 subjects.

Eligibility Criteria. All subjects must:
1. Be Veterans or non-Veterans (male and female; any race; adults (18 & up) of any age).
2. Be English speaking.
3. Be accessible via phone.
4. Be non-decisionally impaired. Determined by assessing the subject's ability to verbalize their understanding of the protocol back to us during the informed consent process.
5. Not have a history of macular degeneration.
6. Not have a history of bipolar disorder.
7. Not be currently using a lightbox or a negative ion generator.
8. Not be a shift worker
9. Must live within the United States during the course of the study.

Every effort will be made to recruit Veterans for the completion of this project. However, to include the recruitment of otherwise healthy subjects without a history of trauma it is necessary to expand recruitment to include non-veterans. The Veteran population is disproportionately male with significant health issues including a history of trauma. Thus, the inclusion of non-Veteran recruitment will help ensure recruitment of more women and underrepresented minorities as well as control subjects without TBI. While we will not specifically target active military personnel for recruitment, we will also include them when they reach out to project staff about participating. Non-Veterans will be notified of specific VHA privacy practices and will be asked to sign the acknowledgment of this notice of privacy practices.

For inclusion in the TBI group:
Subjects must meet the above eligibility criteria and have a self-reported and/or medical record confirmed history of TBI.

Our inclusion/exclusion criteria will not exclude any specific class of persons who might benefit from the proposed research. Every effort will be made to include women and minority groups in this study. All those meeting inclusion/exclusion criteria will be invited to participate in the study, regardless of gender or racial/ethnicity status. Women comprise 8.5% of the Veterans included in the established VAPORHCS Sleep Disorders Data Repository (MIRB#3636) and minorities 8.3%. The same inclusion/exclusion criteria are applied for all participants which ensures that there is no recruitment bias to confound the results.

Power analysis. Pilot data was available for all objective actigraphy outcomes listed in Section 3.5 above, as well as the subjective outcomes of ISI, PHQ9 and Neuro QoL. Literature results by Videnovic et al. [25] provided sham controlled effect sizes for the ESS. To account for the inclusion of the sham control, a placebo effect size was postulated at 50% of the observed MBLT effect with the same variance in outcomes as observed in the pilot cohort. This led to a postulated collection of effect sizes necessary to observe differences in the various outcomes between the MBLT and a sham control. Although some pilot outcomes were observed to have rather extreme effect sizes such as IS (Cohen's d = 1.080, per

arm n = 15) and SE (Cohen's d = 0.375, per arm n = 113), most fell between 0.5 and 1.0, after including a sham control group with postulated, one-treatment per arm sample sizes of 20-70, with a mean sample size of 47 and median of 50 post dropout. This is consistent with prior studies that examined efficacy of MBLT therapy within comparable populations (e.g. depression, dementia), showing effect sizes ranging from 0.20 to 0.90 in sample sizes ranging between n=26 to 34 [26, 32,97]. Given previous published data using CBTi for individuals with TBI showing a dropout rate of 30% [16], an estimated dropout rate of ~20% would yield a per protocol sample size of 24-84 per arm with a mean of 56 and median of 60. Additionally, our previously studies have a 10-15% screen failure. **Therefore, we intend to recruit 100 subjects per group for the lightbox and 50 subjects per group for the negative ion generator.** The 4 groups will be 1) no TBI, lightbox, 2) no TBI, negative ion generator sham, 3), TBI, lightbox 4) TBI, negative ion generator sham (see table below). This sample size should be sufficient to detect clinically meaningful shifts in our outcome variables at levels that are robust enough to be accurately modeled and statistically evaluated.

	TBI	Non-TBI
Lightbox	n=100	n=100
Sham	n=50	n=50

Subject Identification/Recruitment

Primarily Veterans, and some non-Veterans, will be recruited through word of mouth and several other methods:

1. **Repositories:** Participants may be recruited from an existing data repository at VAPORHCS (VA MIRB #3636, PI: Lim, n=670 participants or VA MIRB #4086, PI: Lim, n=200), approximately 30% of whom report TBI. These individuals have all provided informed consent for their data to be kept in the data repository and have given their permission to be re-contacted for future research studies. Existing data on these participants include extensive and detailed history of TBI from the medical chart, severity, and post-concussive symptoms (as assessed by the Rivermead Post-Concussive Questionnaire; RPQ), as well as clinical pain ratings (Likert pain rating scale at multiple time points).
2. **VA and community outpatient clinics: Clinicians will refer eligible patients via VA-encrypted email, over the phone, in person, or by CPRS**, including only name, phone number, and date of visit for follow-up. Consent to be contacted by the study team will be documented on a separate tab in our masterlist on the VA network.
3. **Flyers**: Subjects may be recruited by fliers posted within the VAPORHCS, OHSU, and in the community locally and nationally. In the Portland VA, these flyers will be posted in the building 104 elevator, in handout stands in various clinics, and other VA bulletin boards with approved space for research flyers. They will also be given to clinicians at outpatient clinics to give to interested subjects. Interested subjects who contact us will then be screened over the phone with our phone script. If the subject is eligible and interested after the phone screen, we will plan to meet remotely or in person at VAPORHCS to get written and verbal informed consent. Flyers will also direct potential participants to our study websites as well, and may include a QR code which when scanned directs participants to the websites.
4. **Radio & Web ads:** Subjects may be recruited by short (13-60 second) audio or visual banner or video ads aired by radio stations, using a combination of audio broadcasting and internet streaming as needed to reach more participants. Interested participants will be directed to our lab websites,http://www.portlandsleepstudy.comsharp/ which will direct to which will show our flyers with more information and the number to call. If they qualify, they will come to the Portland VA or remotely undergo the consent process. Additionally, when people stream the radio through a webpage, there will be a link that potential participants can click that will also route to the pvarf webpage with the flyers posted.

5. **Craigslist & institutionally affiliated websites:** including but not limited to OHSU Brain Institute (https://www.ohsu.edu/brain-institute/research-ohsu-brain-institute), USUHS TROOPS ((https://troops.mtbi2.nih.gov), the VA National Center for Rehabilitative Auditory Research (NCRAR) Research volunteers needed webpage: (https://www.ncrar.research.va.gov/Join_Research_Study/Index.asp), and/or other sanctioned venues. Subjects may be recruited by ads on Craigslist may also be used to recruit Veterans as needed. Interested participants must call the number provided; the email option will be disabled on craigslist ads. This recruitment method will only occur at OHSU. The craigslist ad may also direct interested participants to our website www.portlandsleepstudy.com.
6. **VINCI** (VA Informatics and Computing Infrastructure Database): A letter from the study, with an opt-out option may be mailed to potential participants identified through administrative VINCI-based data pulls and electronic review of aforementioned VA outpatient clinics, informing potential subjects of the project. If study staff have not received the opt-out enclosure after 2 weeks, project staff will contact people who were sent letters by telephone to assess eligibility and interest in participation and to arrange an enrollment visit. Study staff will make a total of 3 calls to each listed number before deeming the participant as not interested. We will retain a list of individuals who were deemed not interested (either due to sending the opt-out enclosure or non-response to recruitment calls) so that those individuals are not re-contacted in the future**.** If the participant is eligible and interested after the phone screen, we will meet remotely or in person at VAPORHCS to get written and verbal informed consent.
7. **Research Match**: ResearchMatch.org will be used at OHSU as one of the recruitment tools for this research study/protocol. ResearchMatch Volunteers will be contacted through ResearchMatch.org. Included with this submission is a study recruitment message that will be sent to potential study volunteers. This recruitment method will only occur at OHSU.
8. **OHSU Study Opportunities page:** We will post on OHSU Study Opportunities page and ask that those who are interested to call the number provided.
9. **Portland VA Study Opportunities page:** We will post on the “Join a Research Study at VA Portland” page and ask those who are interested to call the number provided.
10. **SHARP PVARF Website:** As described above, we will have ads that direct participants to our lab’s websites, which will redirect to the Portland VA Research Foundation-sponsored secure website, http://sharp.pvarf.com/join/. Here, information about the studies and flyers will be posted. Additionally, there will be a link to a RedCap prescreening survey where potential participants will answer questions regarding eligibility, and provide their name and phone number if they are interested in being contacted to participate.
11. **Newsletters, press releases, & Social Media Blog Pages:** The Sponsor (Military Traumatic Brain Injury Initiative medicine) would like to post on their blog to advertise for this study nationally. Other VA approved social media or blog features (such as via Direct.gov/GovDelivery or on the US DVA official blog: VAntage Point found at https://blogs.va.gov/VAntage/) may also be utilized, Newsletters and press releases may be pursued in project appropriate venues such as https://www.concussionalliance.org/. These sites may post any version of an IRB approved flyer and/or the IRB approved script and images. Interested participants will be told to call study personnel and/or check out our website.
12. **Sponsor’s TROOPS Referral page:** Potential participants may be referred to this study through the $MTBI^2$ Participant Referral Program, TBI Research Opportunities and Outreach for Participation in Studies (TROOPS). TROOPS is designed to expedite and enhance the participant recruitment and referral process through direct self-enrollment via a web-based platform that is accessible from computers, tablets, and smartphones. All information collected will be voluntarily self-reported by the participants upon their enrollment into the $MTBI^2$ Referral Program, and will be used to determine their eligibility for various $MTBI^2$ studies and $MTBI^2$ collaborative studies. Once referred, the respective study's IRB-approved recruitment and consenting processes will be implemented.

13. **GovDelivery:** GovDelivery delivers email bulletin messages to self-subscribed users. When users register, they select specific topics that interest them. If the recruitment material for our study is deemed to fall within a topic that a given user has subscribed to, they will then receive an email update containing the Public Affairs approved content at the normal frequency for GovDelivery materials. We are not requesting study materials be re-sent to potential participants at a high frequency. GovDelivery will not share the contact or other personal information of subscribers unless a waiver is signed. Users of GovDelivery self-filter the information they receive by email to some extent by choosing which topics they would like to subscribe to updates for. Only potential subjects who have registered to the GovDelivery system of their own accord will receive email updates from the system. Additionally, subjects will only receive update emails when they are deemed to be relevant to the topics the user has chosen to subscribe to. Users have the option to unsubscribe from email updates or alter their topic preferences at any time. Contact information is entered by the user themselves, so the email addresses used by the system are provided by the potential subjects. These addresses are presumably their preferred and correct method of contact. It is not stated that Public Affairs monitors/tracks, as the emails are provided by the subjects themselves rather than a third-party source or existing database.

    In addition, in accordance with the TROOPS registry protocol and with participant consent, the study team may share contact information (name, email, phone number, enrollment status, enrollment date if applicable) of enrolled participants who agree to have their information shared with TROOPS study staff who may contact them to discuss the TROOPS registry in further detail. All PII will be shared in a secure fashion using DoD Safe, Secure ShareFile, encrypted email, or via telephone. Only approved study staff and TROOPs staff will have access to the contact information.

Information about the prescreening survey: The prescreening survey will be a quick survey hosted on OHSU RedCap to collect the names and contact information of potential participants. Before participants complete the survey they will be asked to acknowledge this statement:

“*IMPORTANT NOTICE: the personal information you enter is collected by the Oregon Clinical & Translational Research Institute (OCTRI) at Oregon Health & Science University using Research Electronic Data Capture (REDCap) software. REDCap is password protected and maintained by OCTRI. Any information you share may not be protected under federal law.”*

If they agree they will be brought to a page asking basic eligibility questions like “Are you a Veteran”, “Do you have macular degeneration”, “Do you have bipolar disorder”, “Are you a shift worker”, etc. If the answers to the questions renders them eligible they will be brought to a page to provide their name, phone number, and best time to contact. If they are not eligible they will receive a message saying “Thank you for your interest but you do not qualify for this study at this time. Please check out our other studies listed. Please call (503) 468-6002 if you have questions or concerns. Interested, eligible participants who complete the survey will be contacted by study members in a timely fashion and undergo the phone screen to further assess interest and explain the study.

We may access potential participant’s CPRS record prior to consent in order to determine eligibility and check for behavioral flags. We may search for a diagnosis of bipolar disorder, macular degeneration, and dementia.

<u>Informed Consent & HIPAA Authorization:</u>

Obtaining written informed consent, and HIPAA authorization, will be conducted in English by trained study personnel either remotely, at subject’s homes, or in the VAPORHCS Sleep Disorders Clinic or

other outpatient clinics within the VAPORHCS and will not differ between subjects for any cohort. Prior to obtaining informed consent, subjects will be given a short screening questionnaire to ensure that they meet eligibility criteria. Study personnel will guide subjects step-by-step through the informed consent document and ensure subjects have had all questions answered. Subjects will be reminded several times that participation is entirely voluntary, and they are free to discontinue participation at any point, and likewise, investigators are free to discontinue their participation at any point.

Informed consent and HIPAA authorization may be obtained via phone or video conferencing in one of the following ways:

- *DocuSign* – Study staff will initiate the sending of a DocuSign envelope to furnish the potential participant with links to the consent and authorization forms for conducting the consent conversation. These will be sent with approved template wording that reminds the volunteer not to sign anything until instructed to do so by the consenter during the scheduled phone or video visit. After the consent discussion the consenter will aid the subject in executing the documents and then in retrieving their copies of the fully executed consent and HIPAA Authorization for their records.

  DocuSign will be used as the study's preferred method for fully remote HIPAA/Consent discussion. Choosing to use DocuSign will improve security and privacy for our research volunteers, will alleviate participants' burden in tracking and shipping these crucial study documents, and will help prevent the delays and errors that mail-in consent methods may encounter.

  The study team has been approved by ORD for use of 350 envelopes (emails). Further details are located within the document 'Standard of Operating Procedure: DocuSign for Research'.

- *Mail- (This option will be used in cases where DocuSign is not feasible for any reason.)* Study staff will mail potential participants the consent and authorization forms ahead of time, and then go over them during a video conference. Subjects will sign and mail the forms back to us, and we will sign them when we receive them. We will then mail them copies of the fully executed forms.

  Alternatively, they may sign both the consent and authorization forms, take pictures of each page, and send the images back to us via encrypted email (initiated by study team) or by using MyHealthyVet.

  If taking and sending pictures of each page of the consent and authorization forms is too onerous for the subject we will continue the remote aspects of the visit using a waiver of documentation of consent form. The questionnaires and cognitive testing may take place in this case.

<u>Risks and Side Effects:</u>
The delivery of usual care is never altered by this study. Study risks include breach of confidentiality, psychological discomfort in completing study questionnaires and neuropsychological testing, and mild physical discomfort from MBLT, blood draws, and pressure algometry testing. Participants will be reminded at each event that they do not have to continue with the study questionnaires, pressure sensitivity testing, blood draws, or neuropsychological testing and that they are free to withdraw their consent at any time.

Psychological.

- Questionnaires and Neuropsychological testing: There is a small risk that completing questionnaires could be stressful, discomforting, or mildly embarrassing to the participants. The questionnaires take approximately ~30 min to complete. Since questions are asked about mood, including thoughts of self-harm and depression, there is a possibility that information about thoughts of suicide or evidence of depression will be gained that will require an intervention. How this information will be handled is discussed below in the *Suicidality* section.

Physical.

- Morning Bright Light Therapy: Light from a lightbox is essentially indistinguishable from natural light from the sun and consists of broad-spectrum white light. Morning bright light from a lightbox poses very minimal and rare risks. One study showed that light intensity of 10,000 lux received for an average of 40+ min per day over a five-year period had no major side effects. Minimal and uncommon side effects included eye irritation, irritability, headache, nausea, sensation of glare, dryness of eyes, and dryness of skin (as can occur from natural sunlight exposure). A rare occurrence of mania or rapid-cycling may occur in those with Bipolar Disorder due to too much exposure to bright light. For this reason, this study will exclude those with Bipolar Disorder. The manufacturer notes in the user guide that people with macular degeneration may be more at risk of retinal damage from blue light and should avoid it. Although the lightbox emits very little blue light, those with macular degeneration will be excluded from the study, as stated above in *Inclusion and Exclusion Criteria*. If problems are experienced, participants are free to stop the study at any time and/or call the Study Coordinator with any questions or issues.
- Negative ion generator: The negative ion generator will be modified so that it does not emit negative ions, so there will be no side effects from negative ions.
- Actiwatch: Participants may experience skin irritation from the actigraphy watch wristband, although very uncommon. Participants will be told to remove the actigraphy watch if this occurs. This would not exclude subjects from further participation.
- Blood draws: Participants may also feel some pain from the needle when their blood is drawn. There is a small chance that the needle will cause bleeding, a bruise, or an infection at the draw site. There is a small chance of fainting during blood draws.
- Sweat patch: Wearing the sweat patch may cause mild skin irritation from the adhesive. Subjects will be advised to remove the patch if they are unable to tolerate the protocol.
- Photosensitivity testing. The photosensitivity test is controlled largely by the participant for brightness levels. Standard protocol for using the photosensitivity tester calls for the participant to press a button to indicate when the light level is uncomfortable. The system automatically ceases when determining tolerance to the light. There is a chance of repeated light levels being uncomfortable to the participant in determining the participant's tolerance level. If the participant experiences more than mild discomfort and/or wants to stop the test, the participant may close their eyes and the researcher will stop the photosensitivity test. The risk for triggering a seizure is very low. The potential for inducing seizures is usually with flashes with a frequency > 3Hz.49 The flash rate for our stimulus is ≤ 1Hz. The risk for triggering migraine is very low. Migraines are most often triggered by much faster frequency stimuli (e.g., 60 Hz), and by much brighter light and glare.
- Dim light melatonin assays: There are no reported major risks associated with the home saliva collection protocol. Some minor discomforts may include needing to stay awake a few hours past the subject's intended bedtime in order to complete the sample collection. The dim light conditions necessary for the at home saliva collection could result in an added visual challenge and increased chance of falling. Participants will be instructed to take extra care to minimize tripping hazards and walk cautiously.

Other.

- Because this study collects protected health information, there is always a risk of a breach of confidentiality. This risk is addressed below under *Privacy and Confidentiality*.
- The possible benefits to the participants and to future understanding and improvements in delivery of sleep-related care to Veterans are reasonable and outweigh the risks of minor and rare physical harm, emotional distress, or breach of confidentiality to study participants.

Participant Safeguards:
This study will not include any vulnerable populations. Potential participants will be gauged for their interest in the study prior to being provided a consent form. Those participants that can verbally acknowledge understanding of the study and agree to participate will be consented by trained study personnel. If it appears that the subject is experiencing decisional impairment or an inability to understand the study, such as failing to repeat back the basic concepts of the study, that person will not be permitted to participate.

Suicidality:
In the event that a subject expresses the thought of harming themselves when they are at the clinic for the overnight sleep test, the sleep lab technician or the researcher, if present, will accompany them to the Emergency Department for a warm-transfer.

If during a follow-up phone call the subject expresses thoughts of harming themselves, then the researcher will contact the VA national crisis line at 585-393-7938 or 1-800-273-8255 or the local suicide prevention line at x52857 and connect the subject to that help by phone. In the event a direct phone transfer cannot be completed, then the researcher will ask that the participant remain available by phone. The researcher will provide the VA National Suicide Prevention Hotline with the name and phone of the participant and the VA Hotline will then phone the participant. The researcher will follow-up afterwards with the subject by phone to ensure that the participant has received adequate help and is safe.

In the event that questionnaire answers indicate depression or suicidality, their primary mental health provider will be notified. If they do not have a mental health provider, then their primary care physician will be notified to make a referral. If there is an immediate suicidal ideation, we will walk the participant to the emergency room for a warm transfer.

Benefits:
Participants may directly benefit from this study through the designated morning bright light. Affected outcomes might include improved quality of sleep, an increase in quality of life, improved cognition, and reduction of pain. Subjects will receive modest monetary reimbursement for their time (see *Subject Compensation*).

Protected Health Information:
The following protected health information (PHI) will be collected in this study: name, phone number, email address, dates of research visits, date of birth, address, and last four digits of the social security number. History regarding diagnosis of a sleep disorder; medical history will be confirmed or examined in chart if TBI, PTSD, and/or depression may also be collected. In addition, to creating the GUID for Federal Interagency Traumatic Brain Injury (FITBIR) we will collect name at birth and place of birth (though note that no PHI/PII is ever disclosed to FITBIR). Data will be transferred to FITBIR via the $MTBI^2$ CASA platform. Name, mailing address and full social security number will be shared with Greenphire, in instances where ClinCards is used to issue study payments. At consent, a participant ID code will be assigned to each participant, and this will be used as the identifier for all further data collection. Research questionnaires related to sleep, mood, trauma, and social history will be collected on paper copies (all coded with the unique participant ID number and no identifiable data). The originals will be stored in a locked file cabinet in a locked office in Dr. Lim's VAPORHCS research space. These data will be disclosed to OHSU and stored on OCTRI's HIPAA-secure REDCap database with OHSU as the coordinating site and $MTBI^2$ CASA. Original hardcopy consent and authorization forms will be stored in a

locked drawer and office at the VA in Dr. Lim's research space. Electronic consent and authorization forms will be kept within an approved secure limited-access research server.

PHI that is mailed or transported between a home visit and the VA will also be coded and stored in the locked file cabinet upon receipt.

Prior to consent, first and last name and email address will be provided to DocuSign in order to initiate the electronic consent process. The consent to be used will also be relayed to DocuSign and, for this study, the consent will mention that the subject could have had a TBI, problems sleeping, problems with memory, and/or problems with pain. Please see "Letter_DocuSign Memo for PO" provided with this protocol for details regarding the National VA's decision to deem DocuSign secure and appropriate for e-consent. Screening phone scripts have been developed which include discussion of this transfer of information and ask potential participants permission for transfer. Mail or in person consent is offered as an alternative within these documents.

Multi-Site Study Concerns:
This is a multi-site study involving subject recruitment from both OHSU and VAPORHCS. Recruitment will take place at VAPORHCS, OHSU, and the national community as described in the **Subject Identification/Recruitment** section of this protocol. All study visits will occur remotely, in subjects' homes, or at VAPORHCS. Miranda Lim, is a VA Staff Physician and Research Investigator at VAPORHCS and also a credentialed neurologist at OHSU. She can be contacted by phone at 503-220-8262 x57404, or e-mail at lmir@ohsu.edu. Analysis of biospecimens may take place at $MTBI^2$ or other affiliate institutions or investigators which may include the VA, OHSU, and/or in Dr. Jessica Gill's lab at the NIH.

Resources Available:
This study is sponsored by the Military Traumatic Brain Injury Initiative, formerly known as the Center for Neuroscience and Regenerative Medicine. To address the profound issues related to the diagnosis and treatment of TBI, the United States Congress, through Public Law 110-252, established the $MTBI^2$ as a collaborative intramural program in May 2008. The Congressional Record of May 15, 2008, expressed Congressional intent that the $MTBI^2$ study actual combat casualties cared for at Walter Reed National Military Medical Center (WRNMMC) using advanced neuroimaging technology in collaboration with the National Institutes of Health (NIH). As part of the $MTBI^2$ mission, this study will optimize the scientific advantages of comparing military and civilian cohorts, and position the $MTBI^2$ to transition advances in the field of TBI research from civilian studies to military populations.

Miranda Lim, M.D., Ph.D., is a clinical provider in the Sleep Disorders Clinic at VAPORHCS and a credentialed neurologist at OHSU in the Department of Neurology. There is office space available at the VAPORHCS Sleep Disorders Clinic (building 100-6C), and provided by Research Service (building 101, rooms 432A, 430, 404, and 534) with locked file drawers in a dedicated, locked room (432A). Testing will occur remotely, in subjects' homes, or at the VAPORHCS at the aforementioned rooms available to the study team.

Costs To Subjects:
Participants may incur a minor cost from operating the energy-efficient lightboxes or negative ion generators. The estimated electrical cost to power the lightbox amounts to <30 cents over the course of the study period. There are no other costs involved with participating in the study. Transportation for subsequent visits will not be provided or reimbursed by the study team.

Subject Compensation:

Subjects will receive compensation in gift cards or via ClinCards. Each subject in each group (MBL or negative ion generator) will receive the same compensation.

- Visit 1/2 = $40
    - Subjects will receive $40 in gift cards,via ClinCards or another cash equivalent during either Visit 1 or 2, whichever visit they complete the battery of tests and procedures. Subjects will be given the full $40 whether or not they complete every aspect of the visit.
- Visit 3 = $100
    - Subjects will be given the full $100 in gift cards, via ClinCards or another cash equivalent whether or not they complete every aspect of the visit.
- Visit 4 Follow-up = $40
    - Subjects will be given the full $40 in gift cards, via ClinCards or another cash equivalent whether or not they complete every aspect of the visit.

Total compensation $180

Privacy and Confidentiality:

All physical copies of study documents will be stored in a locked file in a locked room (Dr. Lim's VAPORCHS designated research space). All study related materials will be coded only by participant identification number.

To protect privacy and confidentiality, the electronic master list of study participants and their unique study identification number will be stored on the VAPORHCS limited-access Research drive and protected by password access to the VA computer system This PHI may also be stored on VA OneDrive and other future VA-approved platforms. Only IRB-approved study personnel will have access to this file. Study data, including some PHI such as clinical visit date, will be disclosed to and stored in a HIPAA-secure REDCap database on the OHSU server administered by OCTRI with OHSU as the data coordinating site, as well as through the MTBI^2 CASA platform administered by MTBI^2 and housed at the NIH. De-identified study data will be disclosed to and uploaded onto the FITBIR Informatics System.

Participants will be identified by GUID, the key to which will be accessible only to the investigators. The information gathered during this study will be kept confidential to the extent that the law allows. The subjects will be informed that these results may be published for scientific purposes, provided their identity is not revealed.

Members of the MTBI^2 , Uniformed Services University (USU), Henry M Jackson Foundation (HJF), US Department of Defense (DoD) and NIH, may have access to the study data for auditing purposes.

Information and/or Specimen Management

Study information will be located at both OHSU and VAPORHCS. Information collected from symptom questionnaires at clinical intake will include PHI such as name, mailing address, and phone number on the cover page. Questionnaires will be collected on physical copies with no identifiable data and only a coded participant ID on the cover page. The original hard copy questionnaires, as well as original consent and authorization forms, will be stored in a locked file cabinet in a locked office in Dr. Lim's research space. These data will also be entered into a secure REDCap database housed on the OHSU server.

These original questionnaires, as well as original consent and authorization forms, will be stored in a locked file cabinet in a locked office of VAPORHCS in Dr. Lim's research space. All records will be maintained according to the VA research records retention schedule. The study data will also be entered into a secure REDCap database housed on the OHSU server and MTBI^2 CASA.

The actigraphy watches will be used to collect actigraphy data and will contain sleep patterns, overall light intake, and overall activity levels. The actigraphy watch has no ability to transmit wirelessly and must be connected to a proprietary charging dock via USB read by proprietary software in order retrieve the data. Data will be downloaded onto a password-protected OHSU-networked computer and secured within the HIPAA compliant OHSU OneDrive or OHSU Dropbox. Actigraphy data will be summarized and added to the OHSU REDCap database and MTBI^2 CASA.

HOBOs will be attached to the lightboxes which will track when the lightbox is turned on or off. The HOBOs do not have the ability to transmit wirelessly and must be connected to a USB dock to retrieve data. Data will be downloaded onto a password-protected OHSU-networked computer and uploaded to OHSU OneDrive or OHSU Dropbox.

Audio recordings of neuropsychological testing will be stored on secure, password protected OHSU-networked computer and uploaded to OHSU OneDrive or OHSU Dropbox.

Biological samples will be labeled with the subject's coded ID and date of collection. They will be stored in a -80C freezer on the 5th floor of building 101 and then batch shipped to the NIH for analysis. Saliva samples will be labeled and stored in a -80C freezer, and sent to OHSU or the NIH for batch analysis.

The MTBI^2 GUID is a number assigned by the MTBI^2 Informatics Core. The Informatics Core has established an encrypted system and will provide access to the site for generation of a GUID), developed locally at each site, from personal health identifiers (PHI) data. Only the local site will have access to PHI. Local sites will maintain Master Keys matching GUIDs to PHI. Electronic/computer Master Keys will be kept on password protected terminal(s) in locked rooms with access limited to designated study personnel.

Electronic Master Key records will be backed up electronically at each site at least monthly. Physical print outs/copies of Master Keys will be kept in a locked cabinet in the office of a designated study investigator, and will be updated monthly or at more frequent intervals. The mapping from PHI to GUID will not be stored by or known to the MTBI^2 Informatics Core or NIH CIT personnel, but the central registration of issued GUIDs will help ensure uniformity of identifiers across sites and the ability to identify the enrolling site.

Subjects receiving follow up visits will retain initially-assigned GUIDs throughout their participation and all data will be stored linked to this GUID. Requirements or requests for subject future contact (re-identification) must pass through the enrolling site for GUID-PHI Master Key deciphering. MTBI^2 Master Keys will contain the following information: GUID, last name, last 4 digits SSN, date of birth, and/or medical record number.

The full social security number will be used once to establish participant's initial ClinCard and will not be retained as part of study data.

De-identified study information will be shared outside of VAPORHCS to the FITBIR Informatics System. Depositing the study data is mandatory for all DoD funded studies of TBI. FITBIR does not allow upload of PII, as only approved common data elements that do not include PII can be uploaded.

Data and Safety Monitoring Plan (DSMP)

Participant Safety

Dr. Lim will be responsible for monitoring the safety of the study and complying with the reporting requirements. Continuous, close monitoring of participant safety will include prompt and frequent reporting of safety data (i.e., adverse/serious adverse events) to the local site IRB, the VA, and/or appropriate MTBI^2 staff with oversight responsibility. Serious adverse events will be reported to the local IRB, and the project officer within 48 hours of the time project staff become aware of the incident. All serious adverse events must also be reported to MTBI^2 within 24 hours of site notification. The PI will provide a summary of the safe conduct of the study to project officers on an annual basis as part of the progress report. This report will parallel the written report required by the IRB as part of the annual IRB renewal process. The review of data and procedures may result in early termination of the study, amendment to the protocol, or changes to the data collection plan or study forms. Should the protocol or

data collection plans or study forms be amended as a result of data review, the IRB will be notified and the amendment approved prior to study amendment implementation. In addition, the participants will be notified of any significant new findings that develop during the course of research (e.g., other potential risks) that may affect their wish to continue participation in the study.

It is unlikely that study outcomes will adversely affect the health or well-being of research participants in this experimental study. Participants are under usual care for all treatment they receive for their sleep complaint, in addition to the proposed study arms. If depression or risk of suicide are evident due to questionnaire data collected (e.g., question 9 on the PHQ-9), then the participant will be referred for follow-up. If a participant indicates thoughts of self-harm, then steps will be taken to protect and support the participant as described above in the sections on Suicidality and on Risks and Side Effects.

Data safety and management

Our team has extensive experience collecting and managing research data from human subjects. Our current VA IRB-approved studies operate under a standard operating procedure with specific instructions for administration and entry of questionnaire data. Safeguards are in place to cross-check for duplicate or erroneous data.

Subjects will be assigned a unique subject code as soon as they are consented that will follow them through the study. Inclusion of personally identifiable patient information will be minimized, but some personally identifiable data will be included to decrease errors and allow cross checking of information through, for example, medical chart review. Strict security measures will be used to protect the data, all of which meet or exceed HIPAA requirements. Personal identifiers will only be accessible to authorized study personnel for the purposes for which it was collected.

The goals of our data management system are to maintain data accuracy and security, and to ensure efficient access for monitoring and analysis. The main study database will be maintained within REDCap, administered by the Oregon Clinical and Translational Research Institute. Data can only be accessed using password protection. REDCap performs regular backup of web-based data. This electronic database will eventually contain all data.

All study personnel doing data collection will be carefully trained in their tasks. Collection methods, security, auditing and tracking, prevention and detection of intrusion, back-up and recovery, quality control, monitoring and reporting are briefly described below.

<u>**Location of Research**</u>

VA research: All study visits and blood collection will occur at the VA Portland and will be conducted by staff with VA credentials or WOC appointment. Data analysis will take place at the VA.

Non-VA research: Volunteers will also be recruited outside of the VA at OHSU and in the national community. Recruitment will take place outside the VA as described in the recruitment section of this protocol. Analysis of biosamples will occur at MTBI[2] or other affiliate institutions or investigators such as OHSU and/or in Dr. Jessica Gill's lab at the NIH. Data analysis may take place at OHSU, the NIH, or other affiliate institutions.

Remote visits: All study visits may take place remotely, in which case the location of the research would be the participant's home. This would include use of VA resources (VA video connect, VA equipment), and OHSU resources (RedCap surveys).

<u>**Step-by-Step Guidance on Conducting the Study**</u>

***All aspects of visit may take place remotely with the exception of the blood draw, which must be done in person, at the subject's home, or at the VA. The blood draw does not have to be completed if the sweat patch and saliva collection is completed.**

1. Recruit subjects from methods described in "Subject Identification/Recruitment" section
2. Complete Visit 1 – "Baseline". (May take place remotely)
   a. Obtain written and verbal informed consent and HIPAA authorization from subjects
   b. Collect subjects baseline questionnaire packet and complete pre-study cognition assessment, pre-study light sensitivity assessment, and HTEC interview
   c. Provide subjects with an actigraphy watch and study diary
   d. Provide subjects with saliva tubes and sweat patches
   e. Compensate subjects with $40
3. Allow subjects time to complete their baseline period without MBLT or placebo and schedule time to return their device to the VAPORHCS. Contact subjects regularly via phone to establish rapport and answer questions.
4. Complete Visit 2 – "Pre-study". (May take place remotely)
   a. Provide subjects with light box or disabled negative ion generator
   b. Provide subjects with new actiwatch
5. Subjects use intervention for 1 hour every day when they wake up in the morning. Contact subjects regularly via phone, encrypted email to answer questions.
6. Complete Visit 3 – "Post Study" (May take place remotely)
   a. Collect subjects mid-study questionnaire packet and complete mid-study cognition assessment, mid-study light sensitivity assessment, DLMO salivary collection, sweat patches, and blood draw.
   b. Collect subject's actiwatch, study diary, and device.
   c. Compensate subjects with $100
   d. Debrief subjects about negative ion generator sham condition.
7. Complete Visit 4 - "Follow up". (May take place remotely)
   a. Collect follow up questionnaire packet, cognition assessment, light assessment, sweat patch, and DLMO sample
   b. Compensate subjects with $40
8. Participants who agree to long-term use of data collected in this study will have their study data, blood samples, and contact information from this study added to our existing data repository (IRB#4086).

References & Literature Cited:

1. Hoge, C.W., et al., Mild Traumatic Brain Injury in U.S. Soldiers Returning from Iraq. New England Journal of Medicine, 2008. **358**(5): p. 453-463.
2. Schneiderman, A.I., E.R. Braver, and H.K. Kang, Understanding Sequelae of Injury Mechanisms and Mild Traumatic Brain Injury Incurred during the Conflicts in Iraq and Afghanistan: Persistent Postconcussive Symptoms and Posttraumatic Stress Disorder. American Journal of Epidemiology, 2008. **167**(12): p. 1446-1452.
3. Taber, K.H., D.L. Warden, and R.A. Hurley, Blast-related traumatic brain injury: what is known? J Neuropsychiatry Clin Neurosci, 2006. **18**(2): p. 141-5.
4. Baumann, C.R., et al., Sleep-wake disturbances 6 months after traumatic brain injury: a prospective study. Brain, 2007. **130**(Pt 7): p. 1873-83.
5. Shi, L., et al., Sleep disturbances increase the risk of dementia: A systematic review and meta-analysis. Sleep Medicine Reviews, 2017.
6. Di Meco, A., Y.B. Joshi, and D. Praticò, Sleep deprivation impairs memory, tau metabolism, and synaptic integrity of a mouse model of Alzheimer's disease with plaques and tangles. Neurobiology of Aging, 2014. **35**(8): p. 1813-1820.

7. Nordström, P., Michaëlsson, K., Gustafson, Y., & Nordström, A. . Traumatic brain injury and young onset dementia: a nationwide cohort study. Annals of neurology, 2014. **75**(3): p. 374-381.
8. Julien, J., et al., Association of traumatic brain injury and Alzheimer disease onset: A systematic review. Annals of Physical and Rehabilitation Medicine, 2017. **60**(5): p. 347-356.
9. Plassman, B.L. and J. Grafman, Chapter 44 - Traumatic brain injury and late-life dementia, in Handbook of Clinical Neurology, J. Grafman and A.M. Salazar, Editors. 2015, Elsevier. p. 711-722.
10. Luukinen, H., et al., Head injuries and cognitive decline among older adults: A population-based study. Neurology, 1999. **52**(3): p. 557-557.
11. Mortimer, J.A., et al., Head Trauma as a Risk Factor for Alzheimer's Disease: A Collaborative Re-Analysis of Case-Control Studies. International Journal of Epidemiology, 1991. **20**(Supplement_2): p. S28-S35.
12. Fleminger, S., et al., Head injury as a risk factor for Alzheimer's disease: the evidence 10 years on; a partial replication. Journal of Neurology, Neurosurgery, and Psychiatry, 2003. **74**(7): p. 857-862.
13. Hersi, M., et al., Risk factors associated with the onset and progression of Alzheimer’s disease: A systematic review of the evidence. NeuroToxicology, 2017.
14. Ouellet, M.C. and C.M. Morin, Efficacy of cognitive-behavioral therapy for insomnia associated with traumatic brain injury: a single-case experimental design. Arch Phys Med Rehabil, 2007. **88**(12): p.1581-92.
15. Ouellet, M.C. and C.M. Morin, Cognitive behavioral therapy for insomnia associated with traumatic brain injury: a single-case study. Arch Phys Med Rehabil, 2004. **85**(8): p. 1298-302.
16. Nguyen, S., et al., Cognitive Behavior Therapy to Treat Sleep Disturbance and Fatigue After Traumatic Brain Injury: A Pilot Randomized Controlled Trial. Arch Phys Med Rehabil, 2017. **98**(8): p. 1508-1517.e2.
17. Baddeley, J.L. and D.F. Gros, Cognitive behavioral therapy for insomnia as a preparatory treatment for exposure therapy for posttraumatic stress disorder. Am J Psychother, 2013. **67**(2): p. 203-14.
18. Matthews, E.E., et al., Adherence to cognitive behavioral therapy for insomnia: a systematic review. Sleep Med Rev, 2013. **17**(6): p. 453-64.
19. Gooley, J.J., et al., A broad role for melanopsin in nonvisual photoreception. J Neurosci, 2003. **23**(18): p. 7093-106.
20. Freedman, M.S., et al., Regulation of mammalian circadian behavior by non-rod, non-cone, ocular photoreceptors. Science, 1999. **284**(5413): p. 502-4.
21. Czeisler, C.A., et al., Bright light resets the human circadian pacemaker independent of the timing of the sleep-wake cycle. Science, 1986. **233**(4764): p. 667-71.
22. Czeisler, C.A., et al., Bright light induction of strong (type 0) resetting of the human circadian pacemaker. Science, 1989. **244**(4910): p. 1328-33.
23. Jewett, M.E., R.E. Kronauer, and C.A. Czeisler, Light-induced suppression of endogenous circadian amplitude in humans. Nature, 1991. **350**(6313): p. 59-62.
24. Chellappa, S.L., M.C. Gordijn, and C. Cajochen, Can light make us bright? Effects of light on cognition and sleep. Prog Brain Res, 2011. **190**: p. 119-33.
25. Videnovic, A., et al., *Timed Light Therapy for Sleep and Daytime Sleepiness Associated With Parkinson Disease.* JAMA Neurology, 2017. **74**: p. 411.
26. Tsai, Y.F., et al., *The effects of light therapy on depressed elders.* Int J Geriatr Psychiatry, 2004. **19**(6): p. 545-8.
27. Fonken, L.K., et al., *Dim Light at Night Exaggerates Weight Cain and Inflammation Associated with a High-Fat Diet in Male Mice.* Endocrinology, 2013. **154**(10): p. 3817-3825.
28. Fonken, L.K., Z.M. Weil, and R.J. Nelson, *Dark nights reverse metabolic disruption caused by dim light at night.* Obesity, 2010. **21**(6): p. 1159-1164.
29. Kooijman, S., et al., *Prolonged daily light exposure increases body fat mass through attenuation of brown adipose tissue activity.* Proceedings of the National Academy of Sciences 2015. **112**(21): p. 9748-53.

30. Sakakibara, S., et al., *Effects of morning bright light in healthy elderly women: Effects on wrist activity.* Psychiatry and Clinical Neurosciences, 1999. **53**: p. 235-236.
31. Ancoli-Israel, S., et al., *Increased light exposure consolidates sleep and strengthens circadian rhythms in severe Alzheimer's disease patients.* Behav Sleep Med, 2003. **1**(1): p. 22-36.
32. Riemersma-van der Lek, R.F., et al., *Effect of bright light and melatonin on cognitive and noncognitive function in elderly residents of group care facilities: a randomized controlled trial.* Jama, 2008. **299**(22): p. 2642-55.
33. Dekker, K., et al., *Effectiveness of internet-supported cognitive behavioral and chronobiological interventions and effect moderation by insomnia subtype: study protocol of a randomized controlled trial.* Trials, 2015. **16**: p. 292.
34. LeGates, T.A., D.C. Fernandez, and S. Hattar, *Light as a central modulator of circadian rhythms, sleep and affect.* Nat Rev Neurosci, 2014. **15**(7): p. 443-54.
35. LeGates, T.A., et al., *Aberrant light directly impairs mood and learning through melanopsin-expressing neurons.* Nature, 2012. **491**(7425): p. 594-8.
36. Bowrey, H.E., M.H. James, and G. Aston-Jones, *New directions for the treatment of depression: Targeting the photic regulation of arousal and mood (PRAM) pathway.* Depress Anxiety, 2017.
37. Bedrosian, T.A. and R.J. Nelson, *Timing of light exposure affects mood and brain circuits.* Transl Psychiatry, 2017. **7**(1): p. e1017.
38. Satlin, A., et al., *Bright light treatment of behavioral and sleep disturbances in patients with Alzheimer's disease.* Am J Psychiatry, 1992. **149**(8): p. 1028-32.
39. Golden, R.N., et al., *The efficacy of light therapy in the treatment of mood disorders: A review and meta-analysis of the evidence.* American Journal of Psychiatry, 2005. **162**: p. 656-662.
40. Chesson, A.L., Jr., et al., *Practice parameters for the use of light therapy in the treatment of sleep disorders. Standards of Practice Committee, American Academy of Sleep Medicine.* Sleep, 1999. **22**(5): p. 641-60.
41. Penders, T.M., et al., *Bright Light Therapy as Augmentation of Pharmacotherapy for Treatment of Depression: A Systematic Review and Meta-Analysis.* Prim Care Companion CNS Disord, 2016. **18**(5).
42. Auger, R.R., et al., *Clinical Practice Guideline for the Treatment of Intrinsic Circadian Rhythm Sleep-Wake Disorders: Advanced Sleep-Wake Phase Disorder (ASWPD), Delayed Sleep-Wake Phase Disorder (DSWPD), Non-24-Hour Sleep-Wake Rhythm Disorder (N24SWD), and Irregular Sleep-Wake Rhythm Disorder (ISWRD). An Update for 2015: An American Academy of Sleep Medicine Clinical Practice Guideline.* J Clin Sleep Med, 2015. **11**(10): p. 1199-236.
43. Lam, R.W., et al., *Efficacy of Bright Light Treatment, Fluoxetine, and the Combination in Patients With Nonseasonal Major Depressive Disorder: A Randomized Clinical Trial.* JAMA Psychiatry, 2016. **73**(1): p. 56-63.
44. Martensson, B., et al., *Bright white light therapy in depression: A critical review of the evidence.* J Affect Disord, 2015. **182**: p. 1-7.
45. Nussbaumer, B., et al., *Light therapy for preventing seasonal affective disorder.* Cochrane Database Syst Rev, 2015(11): p. CD011269.
46. Sinclair, K.L., et al., *Randomized controlled trial of light therapy for fatigue following traumatic brain injury.* Neurorehabil Neural Repair, 2014. **28**(4): p. 303-13.
47. Edgar, J.R., *Q&A: What are exosomes, exactly?* BMC Biol, 2016. **14**: p. 46.
48. Anderson, J.L., et al., *Lux vs. wavelength in light treatment of Seasonal Affective Disorder.* Acta Psychiatr Scand, 2009. **120**(3): p. 203-12.
49. Gonçalves, B., et al., *A fresh look at the use of nonparametric analysis in actimetry.* Sleep Medicine Reviews, 2015. **20**: p. 84-91.
50. Kodama, A., et al., *Deriving the reference value from the circadian motor active patterns in the "nondementia" population, compared to the "dementia" population: What is the amount of physical activity conducive to the good circadian rhythm.* Chronobiology International, 2016. **33**: p. 1056-1063.

51. Luik, A.I., et al., *Stability and Fragmentation of the Activity Rhythm Across the Sleep-Wake Cycle: The Importance of Age, Lifestyle, and Mental Health.* Chronobiology International, 2013. **30**: p. 1223-1230.
52. Maaskant, M., et al., *Circadian sleep–wake rhythm of older adults with intellectual disabilities.* Research in Developmental Disabilities, 2013. **34**: p. 1144-1151.
53. Thomas, K.A., R.L. Burr, and S. Spieker, *Maternal and infant activity: Analytic approaches for the study of circadian rhythm.* Infant Behavior and Development, 2015. **41**: p. 80-87.
54. Walsh, C.M., et al., *Rest-activity rhythm disruption in progressive supranuclear palsy.* Sleep Medicine, 2016. **22**: p. 50-56.
55. Whitehead, D.L., et al., *Circadian rest-activity rhythm is altered in Parkinson's disease patients with hallucinations.* Movement Disorders, 2008. **23**: p. 1137-1145.
56. Winkler, D., et al., *Actigraphy in Patients with Seasonal Affective Disorder and Healthy Control Subjects Treated with Light Therapy.* Biological Psychiatry, 2005. **58**: p. 331-336.
57. Castro, J., et al., *Circadian rest–activity rhythm in individuals at risk for psychosis and bipolar disorder.* Schizophrenia Research, 2015. **168**: p. 50-55.
58. Nunes, D.M., et al., *Circadian rest-activity rhythm in chronic obstructive pulmonary disease.* Chronobiology International, 2017: p. 1-5.
59. Sloane, P.D., et al., *High-Intensity Environmental Light in Dementia: Effect on Sleep and Activity.* Journal of the American Geriatrics Society, 2007. **55**: p. 1524-1533.
60. Skjerve, A., et al., *Improvement in behavioral symptoms and advance of activity acrophase after short term bright light treatment in severe dementia.* Psychiatry and clinical neurosciences, 2004. **58**: p. 343-7.
61. Van Someren, E.J.W., et al., *Indirect bright light improves circadian rest-activity rhythm disturbances in demented patients.* Biological Psychiatry, 1997. **41**: p. 955-963.
62. McCurry, S.M., et al., *Increasing walking and bright light exposure to improve sleep in community dwelling persons with Alzheimer's disease: results of a randomized, controlled trial.* Journal of the American Geriatrics Society, 2011. **59**: p. 1393-402.
63. Fetveit, A. and B. Bjorvatn, *The effects of bright-light therapy on actigraphical measured sleep last for several weeks post-treatment. A study in a nursing home population.* Journal of Sleep Research, 2004.**13**: p. 153-158.
64. Saxvig, I.W., et al., *A randomized controlled trial with bright light and melatonin for delayed sleep phase disorder: Effects on subjective and objective sleep.* Chronobiology International, 2014. **31**: p. 72-86.
65. Fiandaca, M.S., et al., *Identification of preclinical Alzheimer's disease by a profile of pathogenic proteins in neurally derived blood exosomes: A case-control study.* Alzheimers Dement, 2015. **11**(6): p. 600-7.e1.
66. Goetzl, E.J., et al., *Altered lysosomal proteins in neural-derived plasma exosomes in preclinical Alzheimer disease.* Neurology, 2015. **85**(1): p. 40-7.
67. Motamedi, V., Kapogiannis, D., & Gill, J., *Exosomal Elevations of Tau and Amyloid-beta Relate to Chronic Symptoms Following a Traumatic Brain Injury*, in *NINR Advisory Council Meeting*. 2016: Bethesda MD.
68. Motamedi, V., et al., *Elevated tau and interleukin-6 concentrations in adults with obstructive sleep apnea.* Sleep Medicine, 2017. **43**: p. 71-76.
69. Gill, J., et al., *Lower health related quality of life in U.S. military personnel is associated with service related disorders and inflammation.* Psychiatry Research, 2014. **216**(1): p. 116-122.
70. Heinzelmann, M., et al., *Sleep restoration is associated with reduced plasma C-reactive protein and depression symptoms in military personnel with sleep disturbance after deployment.* Sleep Medicine, 2014. **15**(12): p. 1565-1570.
71. Livingston, W.S., et al., *Improved Sleep in Military Personnel is Associated with Changes in the Expression of Inflammatory Genes and Improvement in Depression Symptoms.* Frontiers in Psychiatry, 2015. **6**: p. 59.

72. Rusch, H.L., et al., *Improved Sleep Quality is Associated with Reductions in Depression and PTSD Arousal Symptoms and Increases in IGF-1 Concentrations.* Journal of Clinical Sleep Medicine : JCSM : Official Publication of the American Academy of Sleep Medicine, 2015. **11**(6): p. 615-623.
73. Mysliwiec, V., et al., *IGF-1: a potential biomarker for efficacy of sleep improvement with automatic airway pressure therapy for obstructive sleep apnea?* Sleep and Breathing, 2015. **19**(4): p. 1221-1228.
74. Sandsmark, D.K., J.E. Elliott, and M.M. Lim, *Sleep-Wake Disturbances After Traumatic Brain Injury: Synthesis of Human and Animal Studies.* Sleep, 2017. **40**(5).
75. Gilbert, K.S., et al., *Sleep disturbances, TBI and PTSD: Implications for treatment and recovery.* Clin Psychol Rev, 2015. **40**: p. 195-212.
76. Kumar, A. and D.J. Loane, *Neuroinflammation after traumatic brain injury: opportunities for therapeutic intervention.* Brain Behav Immun, 2012. **26**(8): p. 1191-201.
77. Myer, D.J., et al., *Essential protective roles of reactive astrocytes in traumatic brain injury.* Brain, 2006. **129**(Pt 10): p. 2761-72.
78. Kumar, R.G., et al., *Principal components derived from CSF inflammatory profiles predict outcome in survivors after severe traumatic brain injury.* Brain Behav Immun, 2016. **53**: p. 183-93.
79. Devoto, C., et al., *Inflammation Relates to Chronic Behavioral and Neurological Symptoms in Military with Traumatic Brain Injuries.* Cell Transplant, 2016.
80. Kenney, K., et al., *Cerebral Vascular Injury in Traumatic Brain Injury.* Exp Neurol, 2016. **275 Pt 3**: p. 353-66.
81. Chodobski, A., et al., *Early neutrophilic expression of vascular endothelial growth factor after traumatic brain injury.* Neuroscience, 2003. **122**(4): p. 853-67.
82. Mayhan, W.G., *VEGF increases permeability of the blood-brain barrier via a nitric oxide synthase/cGMP-dependent pathway.* Am J Physiol, 1999. **276**(5 Pt 1): p. C1148-53.
83. Weis, S.M. and D.A. Cheresh, *Pathophysiological consequences of VEGF-induced vascular permeability.* Nature, 2005. **437**(7058): p. 497-504.
84. Aston J, D.M., Caraman E, Ballester M, McCarthy M, Olson JE *Brain Edema as a Predictor of Clinical Outcome in Traumatic Brain Injury* in *Research Forum of the American College of Emergency Physicians*. 2008. Chicago, IL: Annals of Emergency Medicine.
85. Morin, C.M., et al., *The Insomnia Severity Index: psychometric indicators to detect insomnia cases and evaluate treatment response.* Sleep, 2011. **34**(5): p. 601-8.
86. Weaver, T.E., et al., *An instrument to measure functional status outcomes for disorders of excessive sleepiness.* Sleep, 1997. **20**: p. 835-43.
87. Johns, M.W., *A new method for measuring daytime sleepiness: the Epworth sleepiness scale.* Sleep, 1991. **14**(6): p. 540-5.
88. Mastin, D.F., J. Bryson, and R. Corwyn, *Assessment of sleep hygiene using the Sleep Hygiene Index.* J Behav Med, 2006. **29**(3): p. 223-7.
89. Kroenke, K., R.L. Spitzer, and J.B. Williams, *The PHQ-9: validity of a brief depression severity measure.* Journal of general internal medicine, 2001. **16**: p. 606-13.
90. Cella, D., et al., *The Patient-Reported Outcomes Measurement Information System (PROMIS): progress of an NIH Roadmap cooperative group during its first two years.* Med Care, 2007. **45**(5 Suppl1): p. S3-S11.
91. Blevins, C.A., et al., *The Posttraumatic Stress Disorder Checklist for DSM-5 (PCL-5): Development and Initial Psychometric Evaluation.* J Trauma Stress, 2015. **28**(6): p. 489-98.
92. Hays, R.D., et al., *Development of physical and mental health summary scores from the patient reported outcomes measurement information system (PROMIS) global items.* Qual Life Res, 2009. **18**(7): p. 873-80.
93. Coffield, T.G. and W.W. Tryon, *Construct validation of actigraphic sleep measures in hospitalized depressed patients.* Behav Sleep Med, 2004. **2**(1): p. 24-40.
94. Marino, M., et al., *Measuring sleep: accuracy, sensitivity, and specificity of wrist actigraphy compared to polysomnography.* Sleep, 2013. **36**: p. 1747-55.

95. De Rui, M., et al., *Sleep and Circadian Rhythms in Hospitalized Patients with Decompensated Cirrhosis: Effect of Light Therapy.* Neurochemical Research, 2015. **40**: p. 284-292.
96. Rissin, D.M., et al., *Simultaneous detection of single molecules and singulated ensembles of molecules enables immunoassays with broad dynamic range.* Anal Chem, 2011. **83**(6): p. 2279-85.
97. Wu, M.C., et al., *The effects of light therapy on depression and sleep disruption in older adults in a long-term care facility.* Int J Nurs Pract, 2015. **21**(5): p. 653-9.

Appendix – Supporting Documents List

Contact info sheet filled out by subjects at consent
Info sheet given to subjects
Demographics form
Questionnaire packet
Cognition assessment
Modified HTEC
Scripts
DocuSign SOPs, Training Materials, ORD approval email, and email template text
Flyers
